# Supplementary material for: Are carbon emissions trading and green financial instruments synergistic? -Comprehensive quantitative research based on content analysis
Source: PLoS One. 2024 Mar 7;19(3):e0298601. doi: 10.1371/journal.pone.0298601 (PMC10919601; doi:10.1371/journal.pone.0298601)
Supplement: S1 Data — (ZIP) [file pone.0298601.s001.zip › Data/地级市Z_F工作报告.zip!/╔╜╢1⁄2/╫═▓⌐╩╨/└┤╫╘╣1⁄2╓┌║┼í╛┬φ┐╦╩2╛▌═°í┐_2015.docx]

各位代表：

现在，我代表市人民政府向大会报告工作，请予审议，并请各位政协委员和其他列席会议的同志提出意见。

一、2014年工作回顾

2014年，全市上下在中共淄博市委的坚强领导下，攻坚克难、积极作为，实现了经济社会持续稳步发展。一年来，重点抓了以下工作：

（一）着力稳增长保发展，经济运行保持在合理区间。积极应对经济下行压力，加强经济运行监测调度，下大气力化解金融风险，提高土地等要素资源保障能力，狠抓重点项目建设，增强“三驾马车”拉动力。全市地区生产总值突破4000亿元，达到4029.77亿元，比上年增长7.4%。粮食总产达到173.6万吨，大旱之年实现“十二连丰”，成功创建为全省出口农产品质量安全示范市。204个重大项目完成投资359亿元，投资亿元以上项目增加34个。固定资产投资、社会消费品零售总额、外贸出口分别增长15.7%、12.4%和6.9%。实现公共财政预算收入292.55亿元，增长7.13%。城市居民人均可支配收入和农民人均纯收入分别增长8.6%和11.5%。

（二）加快转方式调结构，“加减乘”三篇文章向纵深推进。组织实施主导产业“铸链”提升工程，提升优势高新技术产业创新链，全年完成工业技改投资1114.9亿元，淄博被列为全国战略性新兴产业区域集聚发展试点城市，跨入全国城市信息化50强。山东工业设计研究院和齐鲁工业设计创意产业园正式成立。全市高新技术产业产值占比达到30.7%，服务业占比达到40.6%，分别比上年提高1.1个和1.3个百分点。集中力量推动招商引资，面向全国知名民营企业招商取得重大进展，汉能3GW光伏产业集群等重大项目开工建设。成功举办陶博会暨新材料论坛及山东省产学研展洽会。全市实际到位外来投资385.8亿元，同比增长43.2%。

（三）加大节能减排工作力度，生态淄博建设取得新进展。深入开展电力、钢铁、焦化等重点行业综合治理，对316家能耗与排放大户逐一落实综合性治理措施，取缔违法、重污染小企业和加工作坊460家，超额完成黄标车年度淘汰任务，巩固秸秆禁烧和转化利用工作，在全国率先出台煤炭清洁利用监管政府规章，全年煤炭消耗总量净削减超过100万吨，提前一年完成“十二五”节能目标。全市空气质量良好天数增加27天，15个主要河流断面COD和氨氮平均浓度分别改善13.7%和29.3%。下大气力搞好大环境绿化，成功创建为国家森林城市。

（四）坚持统筹城乡发展，新型城镇化迈上新水平。淄博新区建设加快提速，文昌湖旅游度假区开发稳步推进。韩莱路大修、鲁泰大道西延等一批重点工程竣工，南水北调地方配套工程在全省率先运行通水。17个省、市级示范镇开工项目408项、完成投资214.2亿元，四个层级的城镇体系框架逐步完善。成立市国土资源管理委员会和新一届城乡规划委员会，理顺城市管理体制，城乡规划、建设、管理效能不断提高。桓台县被列入全国28个“多规合一”试点。出台全面深化户籍制度改革的意见，推进以人为核心的城镇化。全年开工农房15368户，完成农村公路建设580.6公里，新解决了15万农村人口饮水安全问题。

（五）全面落实民生政策，民生建设取得新成效。全年民生投入占地方财政支出的比重达到57.5%。坚持以创业带动就业，深入实施新一轮“大学生创业引领计划”，新增城镇就业和农业转移人口就业分别完成年度计划的119.7%和106.4%，城镇登记失业率控制在2.73%，保持了就业形势稳定。巩固提升覆盖城乡的全民养老、医疗保障体系，建立大病保险制度，连续十年调增企业退休人员养老金，失业保险、城市低保和农村低保标准分别提高到每人每月900元、450元和每人每年2880元，提高了农村五保和城镇“三无”人员供养标准。提高了基层干部教师待遇。深入推进“十万农户脱贫奔康工程”，年内有4万户、11万人脱贫，我市被列为全国扶贫改革试验区。创新村居审计工作，实现村居审计“制度全覆盖”。启动实施新一轮棚户区改造计划，新开工保障性住房和棚户区改造项目74个、13500套（户），建成10000套（户）。搞好全市社区服务中心布点规划，推行老旧社区简易物业管理，初步解决了脏乱差问题。

（六）加快社会事业发展，保持社会和谐稳定。扎实推进县域义务教育均衡发展和农村义务教育学校校舍标准化建设，在全省率先启动第二期学前教育三年行动计划，促进教育资源均衡配置。深化医药卫生体制改革，与国药集团签署战略合作协议，高青、沂源等全省第二批县级公立医院综合改革试点进展顺利。实施文化惠民工程，“15-20分钟”城乡文化服务圈加速形成，文物保护工作实现重要突破。大力发展体育运动，城镇社区和农村体育健身工程加快推进，我市在第23届省运会上获得总分和奖牌“双第一”。启动实施一孩生育登记及“单独两孩”政策，人口自然增长率控制在4.62‰以内。国防动员和双拥共建工作取得新成效。首创人民防空手语警报，在全国推广普及。深入实施“三四二”社会治理工程，切实加强社会治安、安全生产和食品药品监管工作，全市刑事发案率持续下降。统计、物价、广播电视、民族宗教、外事、侨务、对台、防震减灾、气象、史志、档案、关心下一代、妇女儿童、老龄、慈善、残疾人、红十字、援藏等各项工作都取得了新进展、新突破。

（七）全面深化改革，社会活力明显增强。努力优化营商环境。规范、减少市级行政审批事项51项，在全省率先向社会公布《市级行政审批事项目录》。实施建设项目“联审代办”、市与区县联网在线审批和“一费制”改革，审批时限平均提速30%以上。启动实施工商登记便利化改革，全年新增市场主体3.02万户、注册资本329.8亿元。取消、免征110项行政事业性收费，降低23项收费标准，严格清理整顿经营服务性收费，有效减轻了企业和社会负担。支持齐鲁股权交易中心做大做强，中心挂牌企业达到412家，融资额超过150亿元，有13家企业在新三板挂牌。深化财税体制改革，预决算公开、预算绩效管理、政府购买服务、“营改增”试点等各项改革顺利推进。深化农村产权制度改革，农村土地承包经营权、农村集体土地所有权等5项农村产权确权登记颁证工作扎实推进，地政地籍管理走在全省前列。 政府机构改革基本完成。组建市政府重大行政决策专家咨询委员会和法律顾问委员会，开通淄博市行政执法网，搭建市公共资源交易平台，实现了土地和矿产权网上交易，创建为全国“六五”普法先进城市。

一年来，我们深入开展党的群众路线教育实践活动，不断完善“市长市民面对面”等制度安排，倾听群众呼声，改进政府工作，先后征集社会各界对市政府工作的建议意见2369条，建立长效性制度24项，解决了一大批关系群众切身利益的具体问题，认真践行了为民、务实、清廉的承诺。 我们认真执行市人大及其常委会的决议、决定，自觉接受人大、政协和社会各界监督，主动听取各民主党派、工商联和社会各界意见，办理人大代表建议、政协委员提案474件，代表、委员满意率达99%以上。我们坚持从严治政，坚决惩治腐败，全市纪检监察机关立查案件518件，党纪政纪处分531人。

各位代表，过去一年，全市经济社会发展迈上了新台阶，淄博被列为全国首批生态文明先行示范区，连续两届荣获全国社会治安综合治理优秀市，成功蝉联第四届全国文明城市。在复杂严峻的形势下，能够取得这样的进展，是省委、省政府和市委正确领导的结果，是市人大、市政协与社会各界有力监督和大力支持的结果，是全市广大干部群众团结奋斗的结果。在此，我代表市人民政府，向全市广大干部群众，向各民主党派、工商联、各人民团体和社会各界人士，向中央、省驻淄单位、驻淄部队和武警官兵、政法干警，向海内外所有关心支持淄博改革发展的同志们、朋友们，表示衷心的感谢并致以诚挚的敬意！

在总结成绩的同时，我们也清醒地看到，我市经济社会发展还面临一些突出困难和问题。主要是：经济下行压力较大，企业生产经营困难和潜在风险增多；产能过剩问题比较突出，培育新的增长点任务艰巨；污染治理还不够科学有力，生态环境形势依然严峻突出；保障和改善民生压力加大，社会治理面临许多新课题；营商环境建设与市场主体的要求还不适应，法治政府、服务型政府建设差距较大。对这些困难和问题，我们将逐项深入分析，研定切实有力措施，坚决认真地加以解决。

二、2015年奋斗目标和工作重点

今年是全面深化改革的关键之年，是全面推进依法治国的开局之年，也是全面完成“十二五”规划的收官之年。今年政府工作总的指导思想是：全面贯彻落实党的十八大和十八届三中、四中全会精神以及习近平总书记系列重要讲话精神，坚持稳中求进工作总基调，主动适应经济发展新常态，以提高经济发展质量和效益为中心，进一步丰富“发展、实干”工作内涵，突出改革开放和创新发展，集中力量推进转型升级、生态环保、城乡统筹、文化强市和民生建设，保持经济健康发展和社会和谐稳定，努力在全面建成小康社会进程中走在前列。

今年全市经济社会发展的主要预期目标建议为：地区生产总值增长7.5%左右，公共财政预算收入增长5.7%左右，固定资产投资增长15%左右，社会消费品零售总额增长12.5%左右，进出口总额增长6%左右，城市居民人均可支配收入和农民人均纯收入分别增长9%、10%左右，城镇登记失业率控制在4%以内，全面完成省下达的节能减排约束性目标。在努力完成“十二五”既定任务的同时，认真编制好我市“十三五”规划。

　　　认识新常态，适应新常态，引领新常态，是当前和今后一个时期我国经济发展的大逻辑。这对我们推动老工业城市转型发展提出了新的更高的要求。我们清醒地看到，当前淄博面临的经济下行压力很大，经济结构调整的阵痛前所未有，稳增长、促转调的任务异常艰巨。我们更清醒地看到，经济增速放缓给我市转型升级打开了宝贵的“机会窗口”。新常态不仅是新挑战，更是新机遇。只要我们痛下决心，放下片面追求速度与规模的包袱，彻底摆脱过度依赖资源环境消耗的老路，集中全市人民的智慧与力量，坚定不移地抢抓新机遇、推动转调创，就一定能够加速淄博凤凰涅槃、浴火重生，就一定能够增创新优势、再创新辉煌！多年的努力和探索也使我们更加深刻地认识到，在今年及今后一个时期，要更加注重把握好以下四点：

——进一步突出解放思想，推进深度转调。时刻清醒认识我市发展所处的阶段性特征，正确处理腾笼与换鸟、速度与质效、当前与长远等重大关系，自觉主动地按经济规律办事。抓住机遇，深入实施创新驱动发展战略，调整存量、优化增量，以转求进、以调促优，加快解决结构偏重、核心技术偏少、附加值偏低、能耗和排放偏高的问题，集中精力打造淄博经济升级版。

——进一步突出深化改革，激发市场活力。扎实有序推进各项改革，突出强化企业主体作用，大幅减少政府对资源的直接配置，坚决破除制约市场活力的体制机制障碍，充分释放改革红利，千方百计激发经济内生动力，让全社会创新创业活力竞相迸发。

——进一步突出依法行政，优化发展环境。加快建设法治政府，自觉运用法治思维和法治方式深化改革、推动发展、化解矛盾、维护稳定。务实推进“三最城市”建设，全面优化营商环境，增强区域发展的新活力、新动能。

——进一步突出发展惠民，促进社会和谐。始终抓住发展第一要务不放松，努力实现有质量、有效益、可持续的发展。坚持把群众之所望作为政府工作之所向，下大气力保障和改善民生，让城乡群众共享改革发展成果。

　　　各位代表，面向新的发展，省委省政府作出了“一个定位、三个提升”的部署要求，淄博作为全省发展大局中的重要城市，必须自我加压、积极作为，高点对标、争先进位。要紧紧抓住新常态带来的新机遇，集中力量抓好以下七个方面的工作：

　　　（一）更加扎实有力地稳增长保发展。坚持把稳增长摆在更加突出的位置，确保经济在健康区间运行，为调结构、促改革、惠民生奠定坚实基础。

　　　增强内需拉动力。坚持以优质项目建设为纲不动摇，抓住机遇，策划论证和组织实施一批重点新兴产业项目、一批重点存量调整项目、一批重点基础设施建设项目、一批重点民生和社会事业项目，充分发挥投资的关键性作用。年内集中力量推进首批177个市重大项目，总投资1285亿元。进一步强化“要素资源跟着项目走”的保障机制，实行市和区县领导挂包重大项目责任制，加强全程跟踪服务和专项督查考核，确保重点项目顺利落地开工，尽快投用达效。努力增强消费的基础性作用。引导企业根据消费需求调整产品结构,创新营销模式。加快培育市场空间大、综合效益好的消费热点，创新消费供给，增强消费对就业和经济增长的拉动力。

　　　强化金融支撑作用。务实推进政银企合作，健全市、区（县）两级政策性担保体系和风险补偿机制，解决企业贷款抵质押率偏低、过度依赖担保等突出问题，扩大信贷规模，降低融资成本，加大金融对实体经济的支持力度。抓住资本市场改革发展机遇，提高直接融资比重。高度重视防范和化解金融风险，积极用改革的思路、重组的办法，从根本上破解部分企业资金链困局；全面落实各级政府的主导责任、银行与企业的主体责任，完善风险预警处置、大型客户债权银行联席会议等有效机制，坚决守住不发生区域性金融风险的底线。加强社会信用体系建设，保持打击非法集资和高利贷等违法犯罪的高压态势，维护良好的金融生态环境。

　　　提高财政经济质量。坚持依法治税、应收尽收，不收过头税，确保税收增长真实反映转型升级成果。清理规范财税优惠政策，加快推行税式支出管理，营造法治、公平税收环境。改革财政支持经济发展的方式，清理整合扶持产业发展的有关专项资金，建立政府投资引导基金，放大财政资金杠杆效应。建立“借、用、还”相统一的管理机制，加强政府性债务管控。

　　　（二）更加扎实有力地推进转型升级。结构重、附加值低、污染严重是我市发展面临的最大难题，转方式、调结构必须持久加力，一刻也不能放松。我们必须抓住时机，主动作为，加大力度持续做好“加减乘”促转调三篇文章，努力增创区域竞争新优势。

　　　加快工业由大向强跨越。一是以重点项目为牵引。集中力量抓好100个重点技改项目、60个战略性新兴产业项目，打造24条高新技术产业创新链，集中精力推进国家战略性新兴产业区域集聚发展试点，加快传统产业脱胎换骨，新兴产业倍增翻番。力争到2020年，基本完成增长动力向创新驱动转换。二是以优势企业为支撑。支持骨干企业通过上市挂牌、兼并重组等路径，整合市场优势资源，实现增长补短，努力做大做强，着力解决淄博企业“大而不强，强而不大”的问题，加快培植支撑区域创新发展和产业集群集聚的支柱力量；制定具体措施支持齐鲁石化、山铝、淄矿、新华等中央和省属国有企业深化改革，发展混合所有制经济，集中发展一批技术含量高、投资规模大、环境友好的好项目、大项目；落实支持小微企业发展的政策措施，大力发展民营经济。三是以园区为载体。制定出台全市重点产业功能区建设发展规划、专业化产业基地推进方案和“飞地经济政策”，集中力量培植一批百亿级、千亿级产业集群和特色园区。

　　　大力发展现代服务业。集中抓好总投资750亿元的100个重点项目，力争今年服务业比重再提高1.5个百分点左右。着力打造现代服务业优势品牌：一是打造鲁中商贸物流中心。加强对城市综合体等大型商贸设施的规划引控，构建成熟商圈，加快物流企业整合，改造提升化工、建材、机电等大型专业批发市场，大力发展电子商务，扩大淄博的市场影响力、辐射力。积极创造条件争取淄博保税物流中心提升为综合保税区，抓好齐鲁综合物流港等一批重点项目，改造提升309物流产业带，建设名副其实的鲁中“旱码头”、“无水港”。二是打造鲁中区域性金融中心。着力增强齐鲁股权交易中心的辐射带动功能，争取年内新增挂牌企业100家，融资总额突破200亿元。推动齐商银行引进战略投资者，深化农信社银行化改革，规范发展村镇银行、小额贷款公司。全力支持太平洋在线服务中心等一批重大金融保险项目建设和业务发展，加快淄博新区金融商务区建设，打造淄博金融聚集区。研究制定促进金融产业发展规划和配套政策，做大做强金融产业。三是打造鲁中设计创意中心。加强与清华大学及德国等发达国家顶尖设计机构的合作，建设好、运营好山东工业设计研究院和齐鲁工业设计创意产业园，提高工业设计水平，培育壮大工业设计产业。支持动漫、游戏等创意产业快速发展。积极发展健康、养老、家政等生活性服务业，促进淄博经济轻型化。

　　　加快推进农业现代化。以稳粮增收调结构、提质增效转方式为主线,加快发展现代农业。一是稳定粮食生产。认真做好永久基本农田划定工作，继续抓好粮食高产创建，将粮食播种面积、总产稳定在300万亩和130万吨以上。二是加快发展特色农业。顺应由“生产导向”向“消费导向”转变的大趋势，深入调整农业结构。实施百家市级都市农业园区提升工程和国家果菜茶标准园创建工程，打造一批高效特色农业品牌和专业镇村，推动100万亩优质专用粮、100万亩林果、75万亩蔬菜和黄三角高效生态农业示范区提质增效。三是加快发展生态循环农业。加大农业面源污染治理力度，深入实施秸秆禁烧转化、农药残留治理、重金属污染修复等六大工程，严格保护耕地和水资源，提高农产品质量安全水平。力争农产品标准化生产程度达到70%以上。着眼旱情持续发展的形势，加大措施改善山区水利条件，提高水资源保障能力。四是加快培育农业新型经营主体。每年重点扶持100家龙头企业、50家农民合作示范社和一批示范家庭农场。加快培育新型职业农民。深入实施种业振兴规划，积极推广大型高效复式农机，提高农业现代化水平。

　　　（三）更加扎实有力地改善生态环境。以建设国家生态文明先行示范区为统领，统筹推进节能减排、污染治理和生态修复，力争在天蓝、地绿、水清、气爽上实现突破性进展。

　　　加大节能减排工作力度。严格能耗和污染排放源头控制，坚决卡住新上高耗能、高排放项目，确保完成节能减排目标任务。深入推进能耗和排放大户综合治理，加快淘汰落后产能，坚决取缔污染严重的“土小”企业，集中治理和规范露天开山采石行业。加快调整能源结构，大力开发和推广清洁燃煤技术，依法取缔建成区10吨以下燃煤锅炉及超标排放的燃煤设施，确保年内燃煤消耗总量净削减100万吨以上。深入实施节能减排低碳发展行动方案，全面推进工业节能、绿色建筑、绿色交通计划，研究具体措施支持做大做强节能环保产业。

　　　坚定不移加强环境保护。严格执行国家和省大气污染防治规划及大气污染物排放新标准，搞好中日大气污染防治综合示范区建设，大力推进电力、建陶、矿山等重点行业深度治理，集中整治挥发性有机物和扬尘污染，继续抓紧淘汰黄标车、加油站清理整顿、煤炭质量全程监管等工作，努力增加“蓝天白云、繁星闪烁”的天数。以消除“劣五类”地表水为目标，深入实施污染点源治理再提高工程，建设改造4座城镇污水处理厂，新建6处生态湿地，全面封堵污水直排口，确保主要河流水质稳定达标。建成投运市危险废物集中处置中心、市餐厨废弃物处置厂，开工建设第二环保热电厂。用最严格的标准和最严厉的措施抓好饮用水源地保护，确保环境安全。加大环保公安一体化执法力度，强化基层监管力量，落实“顶格处罚”、“按日计罚”等惩处措施，进一步加强打击环境违法犯罪行为高压态势。

　　　优化城乡生态环境。进一步修订完善功能区划，划定生态红线，建立生态功能保护区。持之以恒抓好森林围城、荒山绿化、农田林网等重点绿化工程，加快提升文昌湖等重点区域绿化水平，突出抓好四宝山、九顶山等生态修复绿化提升工程，建设齐盛湖等一批城市森林绿地，新增造林面积14万亩、园林绿地面积300公顷，巩固提升国家森林城市创建成果。抓好“清水润城”工程建设，着力做好水的文章，扎实推进孝妇河、范阳河、东西猪龙河、乌河等中小河流的综合治理，开工建设孝妇河黄土崖段综合整治工程，加快新区景观水系以及文昌湖、马踏湖、大芦湖、五阳湖、红莲湖、太公湖等生态湿地和绿网建设，构建互联互通、景观宜人的全域生态水系。以矿产资源规划修编为契机，全面优化矿产开发布局，杜绝对生态环境的破坏。抓好路域环境综合整治工程。以治脏治乱治差为重点，集中开展农村环境综合整治，实现城乡环卫一体化全覆盖。首批选取8个镇43个村，实施“美丽乡村连片创建示范行动”，提高生态文明乡村建设水平。

（四）更加扎实有力地推动城乡统筹。充分发挥组群城市优势，大力推进以人为核心的城镇化，提高城乡一体化发展水平。

增强城市辐射带动功能。组织编制城市发展战略规划，调整完善土地利用总体规划，促进“多规合一”，推动“四个层级”协调发展。加快推进淄博新区金融、商务、医疗、教育、文化等功能区块和现代服务业产业园区规划建设，努力建设生态新城、产业新城、宜居新城；加大主城区东南部和区县老旧城区改造力度，配套完善公共设施，进一步提高主城区的核心带动力。促进主城区与次城区在基础设施、产业布局、生态环境等方面优势互补、协调发展。扎实推进文昌湖旅游度假区开发建设。着力抓好以17个省、市示范镇为重点的小城镇建设和农村新型社区建设，积极稳妥地推进空心村搬迁改造，促进农村人口就近就地城镇化。完善市级监督考核、区县具体管理的“扁平化”城市管理体制，努力实现城市全覆盖、全时段精细化管理。通过政府购买服务等方式，进一步健全和提升老旧小区简易物业管理。深入开展城乡环境综合整治，巩固提升国家卫生城市创建成果。

　　　增强基础设施支撑能力。抓住国家加强互联互通基础设施建设的机遇，组织力量积极策划和争取实施一批事关全市长远发展的基础性、战略性重大交通、能源、水利等基础设施项目。今年重点组织实施好济青高铁、济青高速、滨莱高速扩容淄博段工程，晋豫鲁铁路沂源段建设及张东铁路、辛泰铁路电气化中修工程，中石化济青二线淄博段和淄博分输站工程，西电东输特高压工程及济南—高青500千伏超高压输电工程，完善南水北调地方配套续建工程，加快实施小农水重点县、病险水库除险加固、小流域水土保持综合治理等工程。加强公共消防基础设施建设。推进城市供水管网改造，做好抗大旱准备，加快南部山区人畜饮水应急工程建设，保障城乡供水安全。以互联互通和产业融合为重点，积极融入省会城市群经济圈。

　　　加快城乡一体发展。一是推动基础设施向农村延伸。继续抓好农房建设改造、县乡道路改造、农村电网升级、农村饮水安全等重点工程，年内新建农房1.2万户、改造危房1000户，改造农村公路200公里，完成912个村“村村强网”建设任务，再解决16万农村居民饮水安全问题。二是提高农村基本公共服务水平。全面完成农村义务教育学校校舍标准化建设，新建50处农村幸福院，完成60处镇卫生院标准化建设，建设100家影像远程诊断基层工作站，完善提升600个农村文化大院，促进基本公共服务均等化。三是推进农业转移人口市民化。全面落实户籍制度改革意见，吸引有能力、有意愿的农业转移人口转为城镇居民，与城市居民享受平等的公共服务。年内新增城镇户籍人口3万人左右。

　　　（五）更加扎实有力地加快改革开放和创新发展。坚持向改革要活力，向开放要资源，向创新要竞争力，靠改革创新增强区域发展的新动能、新优势。

　　　稳步推进重点领域和关键环节改革。一是倾力打造一流营商环境。坚持建设“三最城市”目标不动摇，进一步深化行政审批制度改革，完善建设项目“联审代办”和“一费制”改革，实行建设项目前置审批区域化评估评审制度和政府购买服务制度，切实提高审批效率，降低服务成本。严格清理整顿涉企收费和垄断经营服务性收费，对行政审批和收费项目实行清单管理，多措并举为企业减负松绑。二是深化农村产权制度改革。搞好承包土地确权登记颁证收尾工作，开展农村集体资产股份制改革试点，建好农村产权流转交易和抵押融资“两个平台”，推动“三农”金融服务创新，深化供销社改革，打通为农服务“最后一公里”。三是深化财税体制改革。认真贯彻执行新《预算法》，构建全面规范、公开透明的预算制度。推进“营改增”扩面试点，落实结构性减税措施。同时，按照中央和省部署，深化其他领域改革。继续推进公共资源交易集中统一规范管理改革，健全完善市公共资源交易平台。有序推进不动产统一登记工作。完善机关事业单位养老保险制度改革，加快事业单位分类改革、收入分配制度改革和公务用车制度改革。

　　　加大力度对外开放。对外开放对于引进优质增量、带动提升存量，具有决定性意义，必须坚定不移。一是坚定不移地扩大招商引资。建立重大招商项目市大班子领导挂包责任制，集中优质要素资源，全力推进与汉能、太保等知名企业的一批重大合作项目，抓紧抓实已签约项目的推进和落地，专业化地搞好重点招商项目策划，更加积极主动地抓好面向世界500强和全国行业100强企业招商，努力实现招商引资的更大突破，力争全年外来优质投资增长30%以上，突破500亿元。加快转变招商引资方式，更加注重引进人才、技术和创新团队，把“给优惠政策”转变为“搭创业平台”和创造国际化工作环境。二是坚定不移地“走出去”。抓住国家加快推进全球布局的战略机遇，充分发挥政府引领和服务作用，以东盟国家为突破口，谋划建设好一批境外、市外“飞地园区”，鼓励有行业比较优势但在本市遇到资源和环境瓶颈制约的企业加快“走出去”。积极支持优势企业实施海外并购，推进淄博宏达矿业等一批境外资源开发项目。三是坚定不移扩大对外贸易。全面落实扩大进出口的政策措施，完善大通关管理体制，加快建设印尼淄博机电产品展销中心等境外营销网络，增加资源能源性产品、先进技术设备和关键零部件进口，提高淄博经济的外向度。

　　　大力实施创新驱动发展战略。全面推进技术创新、管理创新、产品创新、市场创新和商业模式创新，增强转型发展活力。一是加速高新技术产业化。坚持“创新要实”，强化企业的主体地位，建设好新材料等开放式中试基地，支持创新型中小企业利用多层次资本市场上市挂牌融资，大力引进和发展风险投资、创业投资，打通资本市场与创新市场的对接通道，加速科技成果资本化、市场化。二是提高区域创新功能。支持淄博高新区加快建设国家创新型科技园区，完善鼓励高新区创新成果扩散的政策，增强辐射带动功能。建设好技术创新公共服务平台、创新孵化器和创新创业苗圃，提高协同创新、集成创新能力，强化知识产权保护，营造有利于大众创业、万众创新的制度环境和社会氛围。三是强化人才支撑。加强企业家队伍系统培训，建设高素质企业家群体。全面实施淄博英才计划，加强与国家、省外专局的合作，积极引进高层次人才。充分发挥山东理工大学的智力支撑作用，积极推进职业教育校企合作和国际合作办学，为大规模创新发展提供人才保障。

　　　（六）更加扎实有力地建设文化强市。淄博是齐文化的发祥地，要充分挖掘齐文化的深厚内涵和时代价值，繁荣发展文化事业和文化产业，推动文化和经济融合发展，加快建设文化强市。

　　　加强淄博优秀文化资源的挖掘和阐发。坚持社会主义核心价值观取向，组织力量深度挖掘、整理、传播齐文化名人、名著、名器、名址，系统化深刻阐释齐文化尚变革、重开放、崇法治、兼容并蓄、重工崇商的鲜明特质和深厚内涵，通过有扬弃的继承，彰显齐文化的时代价值。整合加强齐文化研究资源，编制齐文化研究开发中长期规划，争取将齐文化研究开发纳入国家和省重点文化发展工程，努力打造富有影响力的区域文化体系和城市文化品牌。深入学习弘扬焦裕禄精神和朱彦夫、孟祥民先进事迹，扎实推进“四德工程”示范区县创建，筑牢全市人民团结奋斗的思想根基。

　　　加快发展文化产业。以齐文化为统领，科学整合聊斋文化、陶琉文化、齐商文化，突出抓好文化与旅游融合发展，加快推进齐古城、稷下学宫、齐故城考古遗址公园、高青陈庄—唐口考古遗址公园等一批齐文化载体项目建设，提升重点景区品位，做大做响淄博文化旅游品牌。大力发展生态文化旅游，加快推动文昌湖、周村古商城、原山、鲁山、潭溪山、牛郎织女等景区的配套提升和名品化发展，重点开发建设淄川太河、博山池上、桓台马踏湖等6个乡村旅游连片发展示范区，打造一批省级旅游强镇和旅游特色村，促进乡村旅游集群化。加快发展陶琉艺术、图书出版、博古文玩、创意会展等新型文化业态，培育一批有影响力的重点文化企业。

　　　加大文化惠民力度。进一步搞好市文化中心建设、管理和运营，不断完善农村、社区文化广场、文化大院等公共文化设施，完善政府购买文化服务长效机制，促进城乡基本公共文化服务均等化。深化国有文艺院团改革，推动优秀剧目走向市场。积极筹备参加山东省第十届文化艺术节。进一步理顺文化执法监管体制。加强文化遗产保护，组织实施“乡村记忆工程”、文物保护重点工程和县以下历史文化展示工程。扶持民办博物馆建设，推动公共图书馆、文化艺术馆、博物馆、美术馆免费开放，大力开展社会科学普及，更好满足群众多元化文化需求。

　　　（七）更加扎实有力地加强民生和社会建设。坚持民生优先，加快发展社会事业，创新社会治理，促进和谐稳定。

　　　统筹做好就业和社会保障工作。一是全面落实扩大就业政策，2014—2018年，每年安排7000万元创业带动就业专项扶持资金；对符合条件的创业人员、小企业分别给予最高10万元和300万元的小额担保贴息贷款，年内发放创业小额担保贴息贷款12亿元以上；加快建设进城务工人员综合服务中心、创业梦工场、创业孵化中心。力争全年新增城镇就业7万人、农村劳动力转移就业7万人，高校毕业生总体就业率达到85%以上。二是加大社会保险扩面征缴力度，促进全民参保登记，稳妥做好城乡居民与企业职工基本养老保险关系转换接续，稳步提高社保、低保待遇水平。三是继续组织搞好“十万农户脱贫奔康工程”，搞好国家扶贫改革试验，开展精准扶贫，力争今年再有2.4万户、6万人整体脱贫。深入推进保障性安居工程，年内开工建设保障性住房2576套、棚户区改造35496套（户），力争用三年左右时间，基本完成全市棚改任务。

　　　推动社会事业改革发展。坚持保基本、补短板、促公平的原则，促进基本公共服务均等化。全面改善义务教育薄弱学校基本办学条件，实施第二期学前教育三年行动计划，促进义务教育均衡发展和普通高中优质特色发展。深化县级公立医院综合改革，加强与国药集团战略合作，加快市中心医院西院区、市公共卫生中心、市妇幼保健院新院区、市中医医院东院区、市残疾人康复中心、乡镇卫生院和村居卫生室提升等重点公共卫生工程建设。稳妥推进“单独两孩”政策，稳定低生育水平。深入开展全民健身运动，提高竞技体育水平，加快培育发展体育产业。大力加强国防动员和双拥共建工作。保障妇女、儿童、老年人合法权益，促进残疾人、慈善、红十字、关心下一代等各项事业健康发展。

　　　加强和创新社会治理。全面推进依法治市，深入开展普法宣传教育，提升社会治理“三四二”格局，提高社会治理法治化水平。健全“党政同责、一岗双责、齐抓共管”的安全生产责任体系，大力推进科技兴安，提高本质安全水平。加强食品药品监管体系和执法能力建设，保障人民群众“舌尖上的安全”。推动信访重心下移，深化涉法涉诉信访改革，运用法治思维和法治方式化解矛盾、维护稳定。深入推进平安淄博建设，完善立体化治安防控体系，建立涉恐涉稳及违法犯罪举报奖励制度，加强基层治保组织建设，推进群防群治。加强舆情收集和舆论引导，营造良好的舆论环境。

　　　三、建设法治政府和服务型政府的主要措施

　　　繁重的改革发展任务和人民群众的新期待，要求我们必须切实转变政府职能，创新行政管理方式，加快建设法治政府和服务型政府。

　　　(一)扎实推进依法行政。各级政府及工作人员要深入贯彻党的十八届四中全会精神，带头尊法学法守法用法，强化法治思维，养成法治习惯，严格按照法定权限和程序行使权力、履行职责，坚决做到法定职责必须为、法无授权不可为。完善依法决策机制，健全重大行政决策专家咨询和法律顾问制度，严格履行法定程序，确保政府决策经得起法律考量和实践检验。全面落实行政执法责任制，健全行政复议和纠错问责机制，切实做到严格、规范、公正、文明执法。认真执行市人大及其常委会的决议决定，自觉接受人大、政协和社会监督，加大政务公开力度，确保权力在阳光下运行。

　　　（二）加快转变政府职能。正确处理政府与市场的关系，按照全省统一部署，在完善行政审批目录清单的基础上，依法制定和公开政府及部门的权力清单、责任清单和市场准入负面清单，充分发挥好市场在资源配置中的决定性作用。突出政府职能向服务型转型，加快完善政府服务平台，倾力打造优质发展环境。充分发挥民生热线作用，提升镇办和村居便民服务中心功能，办好“市长市民面对面”，搭建密切联系群众的桥梁，真心实意为老百姓解难题、办好事。高度关注弱势群体的利益诉求，政策、资金、项目安排优先向困难群体倾斜。完善行政管理相对人评议政府部门制度，真正把评判权交给市场主体和广大群众，倒逼各级各部门更好地转变职能、改进服务。

　　　（三）狠抓廉洁勤政建设。全面落实党风廉政建设责任制，坚决做到党风廉政建设与经济社会发展同谋划、同部署、同落实、同考核。进一步巩固和拓展群众路线教育实践活动成果，严格落实中央八项规定和省市委实施办法，驰而不息纠治“四风”。严格遵守廉政纪律，健全完善惩防并举的反腐败机制，加强对关键领域、关键岗位的监督，以零容忍态度惩治腐败。厉行勤俭节约，反对奢侈浪费，确保“三公”经费只减不增，以为民务实清廉作风取信于民。始终保持奋发有为的精神状态，努力打造一支对党忠诚、个人干净、敢于担当的公务员队伍。坚持把真抓实干、干事创业作为政府工作的“生命线”，不断健全任务明确、执行坚决、督查有力、奖惩科学、问责严厉的落实推进机制，严查领导干部懒政庸政怠政行为，有效解决“为官不为”问题，以踏石留印、抓铁有痕的韧劲和实劲，奋力争取各项工作落地有声、走到前列。

各位代表！淄博正处在转型发展的关键时期，任务艰巨，责任重大。让我们紧密团结在以习近平同志为总书记的党中央周围，在中共淄博市委的坚强领导下，脚踏实地，创新实干，为加快建设文明和谐、富裕秀美的新淄博而奋发努力！
